# Data on dielectric strength heterogeneity associated with printing orientation in additively manufactured polymer materials

**DOI:** 10.1016/j.dib.2018.07.070

**Published:** 2018-08-03

**Authors:** Brad W. Hoff, Sabrina S. Maestas, Steven C. Hayden, Daniel J. Harrigan, Rachael O. Grudt, Michele L. Ostraat, John C. Horwath, Serhiy Leontsev

**Affiliations:** a Air Force Research Laboratory, Kirtland AFB, NM 87117, USA; b Aramco Research Center – Boston, Aramco Services Company, Cambridge, MA 02139, USA; c Air Force Research Laboratory, Wright-Patterson AFB, OH 45433, USA; d UES, Inc., Dayton, OH 45432, USA

## Abstract

The following data describe the dielectric performance of additively manufactured polymer materials printed in various orientations for four common additive manufacturing techniques. Data are presented for selected commercial 3D printing materials fabricated using four common 3D printing techniques: Stereolithography (SLA), Fused Deposition Modeling (FDM), Selective Laser Sintering (SLS), and Polymer Jetting (PolyJet). Dielectric strengths are compiled for the listed materials, based on the ASTM D139 standard. This article provides data related to “Dielectric Strength Heterogeneity Associated with Printing Orientation in Additively Manufactured Polymer Materials” [1].

**Specifications Table**

**Table:** 

*Subject area*	*Materials Science*
More specific subject area	*Dielectric properties of 3D printed polymers*
Type of data	*Table data*
How data was acquired	*High voltage breakdown testing according to the ASTM D149 standard*
Data format	*Raw data*
Experimental factors	*Samples were preconditioned at 23°C and 50% relative humidity for 40 hours before dielectric strength testing*
Experimental features	*These experiments provide dielectric strength data from high voltage breakdown testing of samples printed in various orientations using four common additive manufacturing techniques*
Data source location	*Not Applicable*
Data accessibility	*All relevant data included in this article*
Related research article	Hoff et al. [1].

**Value of the data**

• These data compare dielectric strengths of several different additively manufactured materials printed in various orientations.
• The data highlight anisotropic behavior in additively manufactured materials through the inspection of dielectric properties. Other properties such as mechanical strength, optical properties, and surface properties could be similarly anisotropic.
• The data demonstrate the relationship between processing and performance that may be overlooked in industrial practices and may lead to suboptimal products.
• The data are useful for the design of high voltage insulators based on 3D printed polymers.

## 1 Data

Tabular data previously summarized in Ref. [1] are presented for dielectric strength testing of 3D printed polymers. Dielectric strength testing was performed according to the ASTM D139 standard [2]. Dielectric strength test samples were fabricated using four common 3D printing techniques: Stereolithography (SLA), Fused Deposition Modeling (FDM), Selective Laser Sintering (SLS), and Polymer Jetting (PolyJet) [3]. Data for SLA samples printed using the Watershed 11122 [4] and ProtoGen 18420 [5] polymers, with a layer resolution 0.051 mm, are provided in Table 1, Table 2, respectively. Data for FDM samples printed using the ABS-M30 [6] and ABS-M30i [7] polymers, with a layer resolution of 0.127 mm, are presented in Table 3, Table 4, respectively. Data for SLS samples printed using the DuraForm HST [8] (0.102 mm layer resolution) and Nylon EX [9] (0.152 mm layer resolution) polymers are presented in Table 5, Table 6, respectively. Data for PolyJet samples printed using the VeroBlue [10] and VeroAmber [10] polymers, with a layer resolution of 0.030 mm, are presented in Table 7, Table 8, Table 9 (VeroBlue) and Table 10, Table 11, Table 12 (VeroAmber).

**Table 1:** Dielectric strength data for SLA-printed Watershed 11122 samples.

**Table 2:** Dielectric strength data for SLA-printed ProtoGen 18420 samples.

**Table 3:** Dielectric strength data for FDM-printed ABS-M30 samples.

**Table 4:** Dielectric strength data for FDM-printed ABS-M30i samples.

**Table 5:** Dielectric strength data for SLS-printed Duraform HST samples.

**Table 6:** Dielectric strength data for SLS-printed Nylon EX samples.

**Table 7:** Dielectric strength data for PolyJet-printed VeroBlue (V1) samples.

**Table 8:** Dielectric strength data for PolyJet-printed VeroBlue (V2) samples.

**Table 9:** Dielectric strength data for PolyJet-printed VeroBlue (H) samples.

**Table 10:** Dielectric strength data for PolyJet-printed VeroAmber (V1) samples.

**Table 11:** Dielectric strength data for PolyJet-printed VeroAmber (V2) samples.

**Table 12:** Dielectric strength data for PolyJet-printed VeroAmber (H) samples.

## 2 Experimental design, materials and methods

**Fig. 1:**
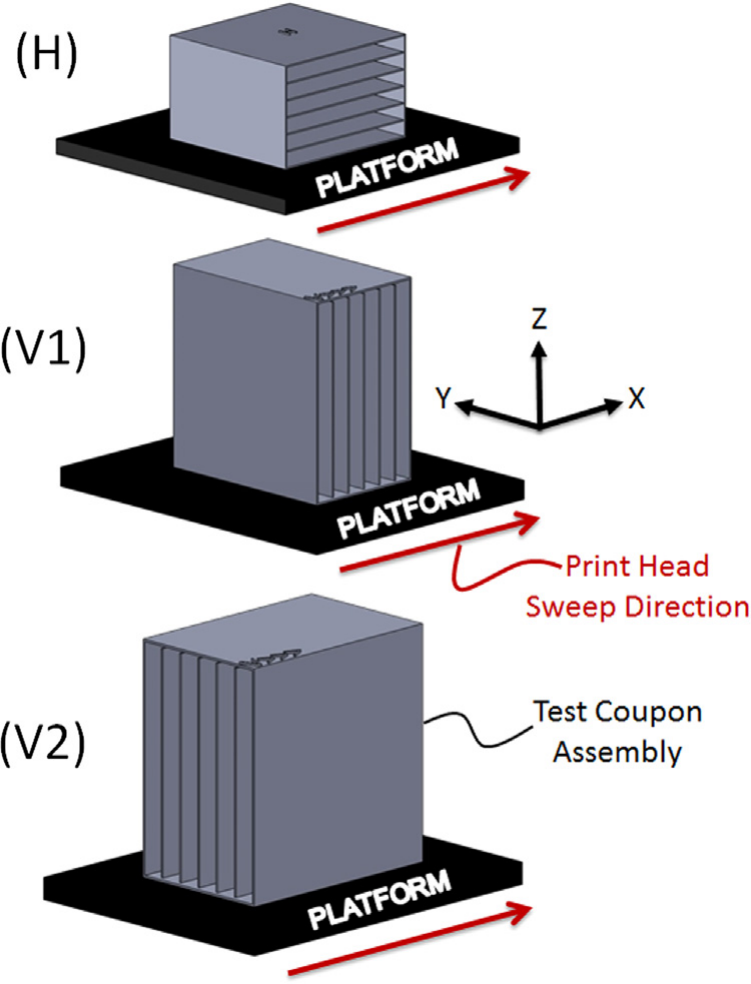
Depictions of sample coupon and shell configurations used in the present study. For printing methods having a well-defined print head sweep direction, such as PolyJet, the sweep direction is indicated by an arrow next to the representative build platforms shown in the figure. Assigning the print head sweep direction to lie parallel to the X-axis follows the convention specified by Ref. [1].

Test sample coupons, fabricated as assemblies in a disposable shell, were printed in either two or three different orientations, as depicted in Fig. 1. For vertically-aligned samples, the surface of each sample face was aligned perpendicularly to the build platform and either perpendicular or parallel to the sweep direction of the print head, as shown in Fig. 1, corresponding to the “(V1)” and “(V2)” designations, respectively. In cases where vertically-aligned samples are fabricated using printing methods in which layer deposition is performed without a print head or nozzle, such as SLS and SLA, it was not expected that there would be a difference in sample properties between vertical configurations (V1) and (V2); as such, these cases are designated only as “(V).” For horizontally-aligned samples, the surface of each sample face was oriented parallel to the build volume, as depicted in Fig. 1. Cases involving horizontally-aligned samples are given “(H)” designations (Table 11).

Upon completion of the printing process, any support materials associated with the printing process that were in the regions between test coupons or otherwise attached to the assembly were removed. As part of the standard manufacturer printing protocol, all SLA-printed parts were UV post-cured for one hour. Post cure procedures are potentially available for other printing methods; however, as they are not standard protocol, they were not performed for this study.

**Fig. 2:**
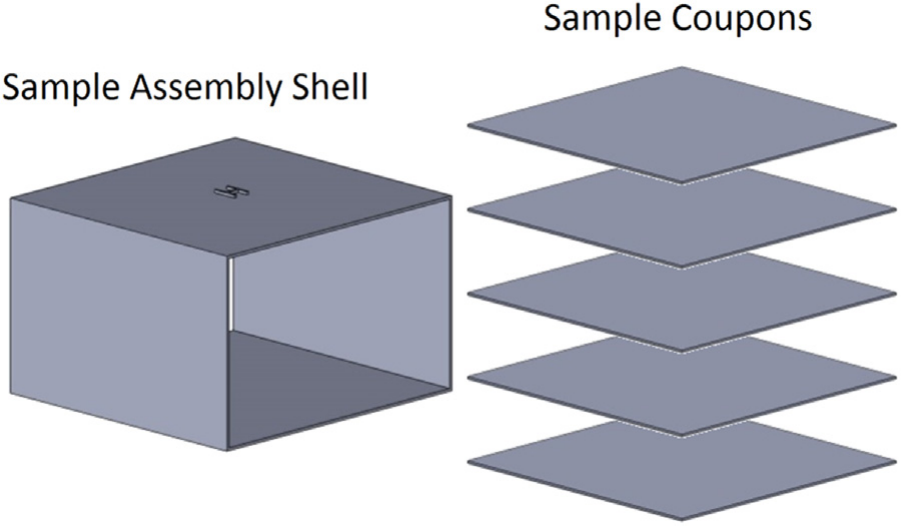
Schematic representation of the sample assembly shell and sample coupons.

In preparation for high voltage testing, each of the sample assemblies was separated into five sample coupons (101.6 mm × 101.6 mm × 1.0 mm) and a disposable protective shell, as shown in Fig. 2. After separation, each sample coupon was cleaned via gentle abrasion while immersed in Liquinox (*aq.*, 1% solution). Following six rinses with deionized water, sample coupons were placed between sheets of lint-free tissue and allowed to air dry at ambient temperature. Dry coupons were stored between clean sheets of lint-free tissue in a desiccated environment for transportation to the dielectric strength testing laboratory.

Just prior to testing, the sample coupons were pre-conditioned for 40 hours at 23 °C and 50% relative humidity. All coupons were tested per ASTM D149-09 (2013), Paragraph 12.2.1, Method A (short time test) [2] using 2.54 cm diameter stainless steel electrodes (ASTM “Type 2” electrodes) in a transformer oil bath. A voltage ramp rate of 500 VAC, RMS (60 Hz)/second was used. Ambient room conditions during testing were approximately 23 °C and 50% relative humidity.
